# Exploring the potential mechanism of Fritiliariae Irrhosae Bulbus on ischemic stroke based on network pharmacology and experimental validation

**DOI:** 10.3389/fphar.2022.1049586

**Published:** 2022-11-17

**Authors:** Wang Zijie, Jiang Anan, Xiao Hongmei, Yuan Xiaofan, Zhang Shaoru, Qin Xinyue

**Affiliations:** Department of Neurology, The First Affiliated Hospital of Chongqing Medical University, Chongqing, China

**Keywords:** fritiliariae irrhosae bulbus, ischemic strokes, network pharmacology, molecular docking, animal experiment

## Abstract

**Objective:** To study the potential targets and molecular mechanisms of Fritiliariae Irrhosae Bulbus (FIB) in the treatment of ischemic strokes based on a network pharmacology strategy, with a combination of molecular docking and animal experiments.

**Methods:** The active components and targets of FIB were screened by TCMSP database and TCMIP database, and the related targets of ischemic strokes were screened by GeneCards, OMIM, CTD, and DrugBank, then the intersection targets of the two were taken. The protein interaction network was constructed by STRING, the PPI network diagram was drawn by using Cytoscape software, and the key targets of FIB treatment of ischemic strokes were analyzed by MCODE. The DAVID database was used for GO and KEGG enrichment analysis, and the potential pathway of FIB against ischemic strokes was obtained. Molecular docking was performed by using AutoDock Tools 1.5.6 software. Finally, a mouse model of ischemic stroke was established, and the results of network pharmacology were verified by *in vivo* experiments. Realtime Polymerase Chain Reaction was used to detect the expression levels of relevant mRNAs in the mouse brain tissue. Western blot was used to detect the expression levels of related proteins in the mouse brain tissue.

**Results:** 13 kinds of active components of FIB were screened, 31 targets were found in the intersection of FIB and ischemic strokes, 10 key targets were obtained by MCODE analysis, 236 biological processes were involved in GO enrichment analysis, and key targets of KEGG enrichment analysis were mainly concentrated in Neuroactive light receptor interaction, Calcium signaling pathway, Cholinergic synapse, Hepatitis B, Apoptosis—multiple specifications, Pathways in cancer and other significantly related pathways. There was good binding activity between the screened main active components and target proteins when molecular docking was performed. Animal experiments showed that the infarct volume of brain tissue in the FIB treatment group was considerably reduced. RT-qPCR and the results of Western Blot showed that FIB could inhibit the expression of active-Caspase3, HSP90AA1, phosphorylated C-JUN, and COX2.

**Conclusion:** Based on network pharmacology, the effect of FIB in the treatment of ischemic strokes was discussed through the multi-component-multi-target-multi-pathway. The therapeutic effect and potential mechanisms of FIB on ischemic strokes were preliminarily explored, which provided a ground work for further researches on the pharmacodynamic material basis, mechanism of action and clinical application.

## 1 Introduction

Stroke is an acute cerebrovascular disease caused by sudden rupture of cerebral blood vessels or vascular obstruction leading to ischemic and hypoxic damage of brain tissue which can be divided into hemorrhagic stroke and ischemic stroke. Ischemic strokes account for approximately76% of all strokes (Chung et al., 2014; Virani et al., 2021). The research findings of two articles published in *The Lancet* in 2019 and 2021 presented that between 1990 and 2019, stroke is the second leading cause of death and the third leading cause of disability in the global population; meanwhile, the first cause of death in China (Zhou et al., 2019; Ward et al., 2021). It follows that the prevention and treatment of global stroke disease is not optimistic, and clinically, we still confront a large number of challenges. Intravenous thrombolysis is one of the most effective measures for early recovery of cerebral blood flow, saving brain tissue and decreasing the incidence of disability. Due to the narrow time window and the risk of hemorrhagic transformation, the proportion of patients who actually receiving vascular recanalization treatment is quite low. A study in 2022 showed that the intravenous thrombolysis rate in China is 5.64%, and the intravascular treatment rate is only 1.45%. More than 92% non-thrombolytic patients with cerebral infarction in clinic lack effective drugs (Ye et al., 2022). Therefore, how to effectively enhance the therapeutic effect of ischemic stroke has become a challenge for clinical and scientific researchers.

Since 2018, numerous evidence-based practice guidelines for treatment of stroke with integrated traditional Chinese and western medicine in China have recommended the combination of traditional Chinese medicine treatment on the basis of conventional treatment of ischemic stroke (Liu L et al., 2020). According to the textbooks of traditional Chinese medicine published by the Ministry of Education of China, plenty of types of ischemic stroke can be classified as heat syndrome, such as the reduction of qi and blood, increase of wind and fire (Shia et al., 2011; Tsai et al., 2013). Thus, traditional Chinese medicines for heat-clearing and fire-clearing are often used in the treatment of ischemic stroke. Fritiliariae Irrhosae Bulbus (FIB) is a dry bulb of various plants of the genus Fritillaria in the Liliaceae family (Chen et al., 2019)which has pharmacological activities, with heat-clearing, phlegm-resolving, anti-inflammatory, reducing swelling, antibacterial, antiviral, and antioxidant functionsIt is also widely used clinically to lower blood pressure, control blood glucose, and prevent and treat recurrent asthma (Sun et al., 2013; Liu S et al., 2020; Zhao et al., 2020). So far, there is few researches on the mechanism of FIB’s target on nerve cells.

**FIGURE 1:**
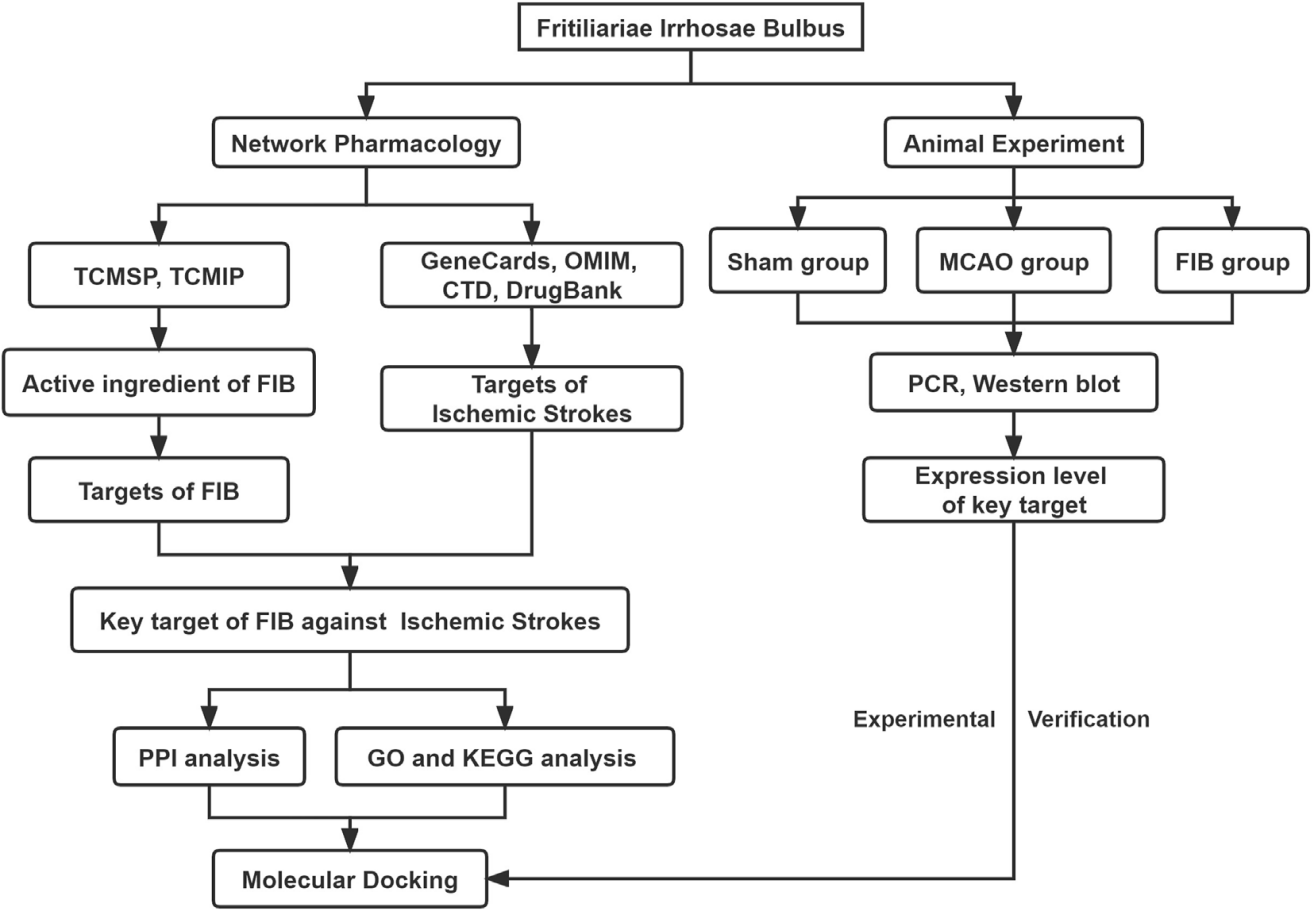
Flow chart of the network pharmacology of Fritiliariae Irrhosae Bulbus (FIB) against Ischemic Strokes.

Although traditional Chinese medicine is extensively used, and still forbidable to elucidate the mechanism of action at the molecular level through experiments due to its “multi-component, multi-target” characteristics, which awfully hindered the modernization of traditional Chinese medicine research. The emergence of network pharmacology provides a new method for identifying specific targets and their interactions of active compounds in traditional Chinese medicine based on molecular mechanisms (Zhou et al., 2020). Network pharmacology analysis utilizes the integration of massive network database resources, chemical informatics and bioinformatics technologies to construct a whole network of drugs and disease targets, thereby guided by the basic and clinical research of traditional Chinese medicine. The holistic and systematic characteristics of the methods coincide with the principles of integral and dialectical treatment of traditional Chinese medicine, especially for recognizing targets, predicting indications, and explaining the mechanisms of action of traditional Chinese medicine (Luo et al., 2020; Li, 2021). In order to further standardize and guide the research of network pharmacology, the Professional Committee of the Network Pharmacology Committee of World Federation of Chinese Medicine Societies prepared the first international *Guidance of the Network Pharmacology Evaluation Method* in 2021, which is the first international standard specialized in network pharmacology evaluation (Lyu et al., 2017). Furthermore, molecular docking is a theoretical approach to study the interaction and recognition between small molecule ligands and protein receptors. Combining network pharmacology and molecular docking technology to jointly study traditional Chinese medicine for stroke, provided a new method for screening active ingredients and mechanism exploration (Villoutreix et al., 2008; Vakser, 2014). Hence, this study aims to frame the interaction pathway between diseases and drug targets through network pharmacology, verify the interaction relationship between core targets and main components by molecular docking technology, explore the mechanism of FIB on ischemic stroke, and confirm it in animal experiments (Figure 1).

## 2 Materials and methods

### 2.1 Screening FIB components and predicting targets

The active chemical components of FIB were collected through Traditional Chinese Medicine Systems Pharmacology Database and Analysis Platform (TCMSP, http://tcmspw.com/index.php) and Integrative Pharmacology-based Research Platform of Traditional Chinese Medicine (TCMIP, http://www.tcmip.cn/TCMIP/index.php/Home/Login/login.html). According to the oral bioavailability (OB) of the drug ≥30% and the drug similarity (DS) ≥0.18, the active ingredients of FIB were further screened out (Liu et al., 2022). According to the two databases, we have integrated the targets involved in the active ingredients, and performed the comparison of target information and target name correction through the UniProt database (https://www.uniprot.org), then made a conversion of the targets into a unified gene name, defining the source of the gene to “*Homo sapiens*”.

### 2.2 Collection of ischemic strokes disease targets

We have utilized disease databases GeneCards (https://www.genecards.org/), OMIM (http://omim.org/), CTD (https://ctd.mdibl.org/) and DrugBank (https://go.drugbank.com/) database, taken “Ischemic Strokes” as retrieval termto search and collect the targets of ischemic strokes, and then, finally obtained the relevant targets of ischemic strokes. The targets obtained from the GeneCards database were classified by the correlation scores of the results, and filtered by using the median score>2-fold as the screening condition. After combining the genes of the four databases and removing the duplicate values, the result of relevant target of ischemic strokes will be showed.

### 2.3 Intersection of ischemic strokes-related targets and FIB potential targets

The online tool Venny2.1.0 (https://bioinfogp.cnb.csic.es/tools/venny/index.html) was used to map mutually the action target of FIB and the target of ischemic strokes related genes and acquire the intersection target of the two namely, the potential target of FIB treatment of ischemic strokes.

### 2.4 Construction of Traditional Chinese Medicine-Active ingredient-target interaction network

The network diagram of “Traditional Chinese Medicine-active ingredients-target” was constructed by using the Cytoscape 3.7.2 software. The nodes represent the drugs, drug components, and target genes in the network, and the edges represent the interactions between nodes. The degree of a node is related to the number of edges which connected to the node. The larger the degree centrality (DC), the more nodes in the network related to the node directly, and then the more important the node in the network (Liu et al., 2022).

### 2.5 The construction and module analysis of protein-protein interaction network (PPI)

The screened common target genes were analyzed using the STRING (https://string-db.org) online database platform; the species was set to “*Homo sapiens*”, and the confidence level was set to ≥0.4 to construct a network diagram of protein interaction between drugs and diseases. Then, the data information obtained from STRING platform was imported into the Cytoscape 3.9.1 software for analysis, sorted according to the degree value (degree), and filtered according to the degree ≥ median to obtain the core target of FIB treatment of ischemic strokes. Molecular complex detection (MCODE) is applied to identify the characteristics of closely connected networks and further screened the core target community. The setting parameters are as follows: find clusters = in the whole network, degree cutoff = 2, node score cutoff = 0.2, K-score = 0.2, max depth = 100 (Khan and Lee, 2022).

### 2.6 GO and KEGG enrichment analysis

Based on DAVID 6.8 database (https://david.Ncifcrf.gov/), gene ontology (GO) function annotation and Kyoto Encyclopedia of Genes and Genomes (KEGG) pathway enrichment analysis were carried out for the intersection targets to obtain the biological process, cell components, molecular functions and related signaling pathway relationships of FIB in the treatment of ischemic strokes. The screening standard was set as *p* < 0.05, and the species were limited only to *Homo sapiens*. GO and KEGG enrichment analysis were made by R language software for visualization.

### 2.7 Molecular docking

Molecular docking is performed between the top 4 active pharmaceutical ingredients and the core targets in the degree value obtained after the analysis. Utilizing the PDB database (http://www.rcsb.org) to retrieve receptor proteins Caspase3 (PDB: 7RNB), PTGS2 (PDB: 3NT1), JUN (PDB: 6Y3V), and HSP90AA1 (PDB: 4BQG) which those receptor proteins will be performed to remove water and ligands by using PYMOL 2.3.4 software The PubChem database (https://pubchem.ncbi.nlm.nih.gov) is used to obtain the SDF structure file of the compound, and AutoDockTools 1.5.6 software is used for hydrogenation, electronation and other operations. AutoDock software is used for molecular docking, and the PyMOL software for data visualization.

### 2.8 Materials

FIB (NO: DZYC-C-022) was purchased from Chengdu Herbpurify Co., Ltd. (Chengdu, China) and dissolved in Carboxymethyl cellulose (Shanghai Aladdin Biochemical Technology Co., Ltd., Shanghai, China). 2,3,5-Triphenyl Tetrazolium Hydrochloride (TTC) staining solution and BCA protein concentration determination kit were purchased from Beijing Soleibo Technology Co., Ltd. (Beijing, China). The monofilaments were purchased from Beijing Cinontech Co., Ltd. (Beijing, China). RNAeasy™ Animal RNA Isolation Kit with Spin Column and Reverse Transcription Kit were purchased from Beyotime Biotechnology Co., Ltd. (Shanghai, China). Specific primary antibodies (Caspase3, Active-caspase3, COX2/PTGS2, c-JUN, phosphorylated-c-JUN, Hsp90α) and HRP Goat Anti-Rabbit IgG (H + L) were purchased from Wuhan ABclonal Technology Co., Ltd. (Wuhan, China).

### 2.9 Transient middle cerebral artery occlusion (tMCAO) model

The animal experiment was approved by the Institutional Animal Care and Use Committee (IACUC) of the First Affiliated Hospital of Chongqing Medical University. Male C57BL/6J mice weighing 20–22 g were provided by the Animal Research Center of Chongqing Medical University, and placed in an environment free of specific pathogens, temperature (22 ± 2)°C, and relative humidity of 50%–70% and 12 h light/12 h dark cycle with free access to food and water. Experiments were carried out after 1 week of adaptive feeding. Before surgery, the fasted mice for 12 h, had free access to drink water, and were anesthetized with 3% pentobarbital. Establishment of middle cerebral artery occlusion or reperfusion model by wire embolization (Luo et al., 2018). After disinfection, cut the skin along the middle of the neck to expose the right common carotid artery (CCA), internal carotid artery (ICA) and external carotid artery (ECA). The proximal end of the CCA and the distal end of the ECA were ligated respectively, and a small slit was cut in the CCA to insert the monofilaments from the CCA into the ICA. After a little resistance, the monofilaments were stopped, and fixed with a silk knot above the CCA incision. After 60 min of ischemia, the monofilaments were removed, and then the live knot on CCA was fastened for reperfusion.

### 2.10 Grouping of experimental animals

Eighteen experimental animals were randomly divided into the following groups (*n* = 6): sham operation group, MCAO group, FIB treatment group. FIB was dissolved in 0.5% carboxymethyl cellulose, and 3 h after reperfusion, 20 mg/kg was administered by gastric perfusion for 5 consecutive days. The sham operation group and the MCAO group were given the same volume of normal saline by gastric perfusion for 5 days.

### 2.11 Neurological deficit score

The Modified Neurological Severity Scores (mNSS) was used for analysis (Ke et al., 2022). The mice were scored on the first, third and fifth days of modeling to observe the neurological deficit of mice in each group, including exercise test, sensory test, balance test, reflex test and abnormal exercise. There was 18 points in total which means the higher the score, the more serious the neurological deficit.

### 2.12 2,3,5-Triphenyl Tetrazolium Hydrochloride (TTC) staining

After 5 days of reperfusion in mice, mice were killed by the overdose anesthesia, brain tissue was taken out and placed at - 20°C. After 30 min, take out the brain tissue and cut it into 6 pieces, and incubate it in TCC solution for 30 min at 37°C. The infarct size was calculated by ImageJ (version 1.37C; Bethesda, United States).

### 2.13 Real-time quantitative PCR

The total RNA from murine brain tissues were extracted with RNA extraction kit, reverse transcribed into cDNA template with reverse transcription kit. BIO-RAD fluorescence quantitative PCR was used for amplification, GAPDH was selected for standardization, and 2^−△△Ct^ method was performed to calculate the relative expression of genes (Livak and Schmittgen, 2001). Primers were purchased from Tsingke Biotechnology Co., Ltd. (Beijing, China).

The primer sequences are as follows: GAPDH: F 5′CAA​AGC​AGG​GAG​TTT​AGT​C3′ R 5′GTG​AGA​TGG​CTC​TGA​GTG​T3′. Caspase-3: F 5′-AGT​GGG​ACT​GAG​GAG​AT-3′, R 5′-GTAACC AGG​TGC​TGT​GAG​GTA​AG-3’. COX2: F 5′-AAT​GAG​TAC​CGC​AAA​CGC​TTC​T-3′, R 5′-TTC​TGC​AGC​CAT​TTC​CTT​CTC-3’. C-JUN:F 5′-ACG​ACC​TTC​TAC​GAC​GAT​GC-3′, R 5′-AGT​TGC​TGA​GGT​TGG​CGT​AG-3’. Hsp90α:F 5′-AGA​CGC​GCT​CCT​TTT​GAT​CT-3’, R 5′-TTG​TTG​CAG​CAT​TTC​ACG​GG-3’.

### 2.14 Western blot

After modeling, the brain tissues of mice in each group were collected, the blood stains were washed with pre-cooled PBS solution, and RIPA lysis buffer was added to extract proteins in a certain proportion. After centrifugation, the supernatant was collected, the protein concentration was measured using a BCA kit. Proteins were separated by SDS-PAGE. The samples were transferred to PVDF membranes and blocked with 5% nonfat dry milk for 1 h. Primary antibodies (Caspase 3, Active-caspase 3, COX 2/PTGS2, p-c-JUN, c-JUN, Hsp90α) were incubated overnight at 4°C, washed 3 times with TBST, and incubated with corresponding secondary antibodies at 37°C for 1 h. Finally, the protein bands were imaged and analyzed using the Gel imaging analyzer system (version GelDoc GO; BIO-RAD, United States), and the value of *p* < 0.05 was considered statistically significant by using the Western Blot imprinted membrane-gel imaging system.

### 2.15 Statistical analysis

Statistical analysis was performed with SPSS 24.0 software. The results have been represented as $\bar{x}\pm s$ . *t*-test is used for comparison between two groups, and One-Way ANOVA test is used for comparison among multiple groups. All data were from at least 3 independent experiments. *p* < 0.05 were considered statistically significant.

## 3 Results

### 3.1 Potential targets of FIB in the treatment of ischemic strokes

**FIGURE 2:**
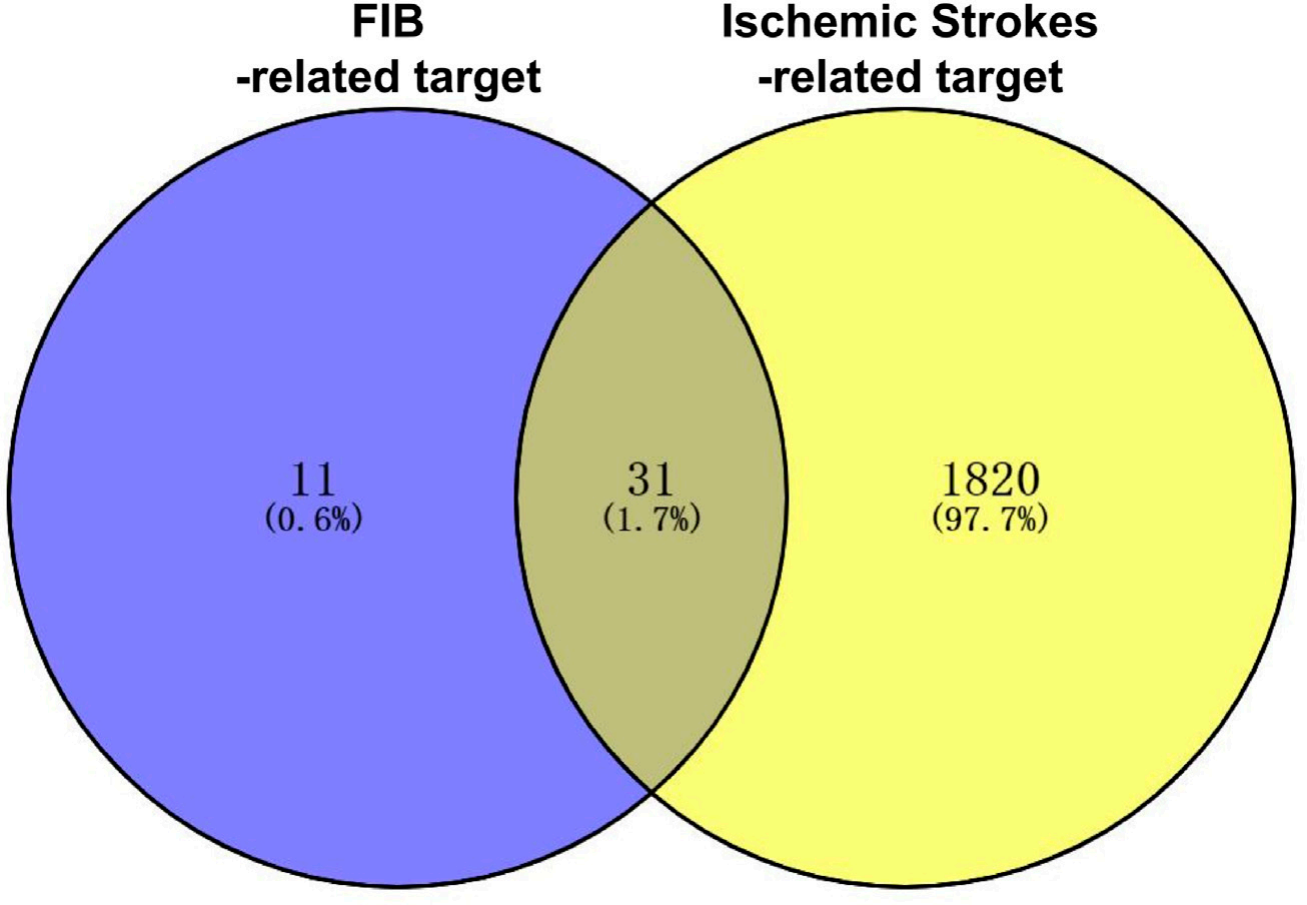
The 31 overlapping targets of FIB and Ischemic Strokes were identified by a Venn diagram.

After retrieving through TCMSP and TCMIP databases, screening according to OB and DL conditions, and deleting duplicate values, a total of 13 active ingredients were obtained, as shown in Table 1. The target proteins correspond to the active ingredients collected by two databases, after deleting the same targets, finally we have obtained 61 targets. The active ingredients mainly included bis [(2R)-2-ethylhexyl]benzene-1,2-dicarboxylate, beta-sitosterol, Korseveriline, verticinone, etc. The target results of ischemic strokes collected from Genecard database, OMIM database, CTD database and Drugbank database were merged, and then the duplicate values were removed, finally 1850 target points of ischemic strokes related gene diseases were obtained. 61 FIB drug targets and 1850 ischemic strokes related gene targets were mapped with online tools Venny2.1.0 to obtain 31 intersection targets for FIB treatment of ischemic strokes (Figure 2).

**TABLE 1:** 13 active compounds of Fritiliariae Irrhosae Bulbus.

Mol ID	Molecule name	MS	MW	OB (%)	DL
MOL001749	bis [(2R)-2-ethylhexyl] benzene-1,2-dicarboxylate	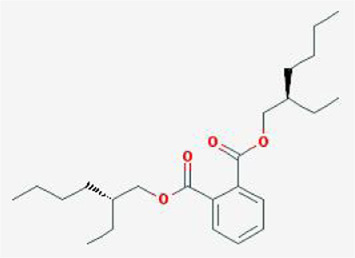	390.62	43.59	0.35
MOL000358	beta-sitosterol	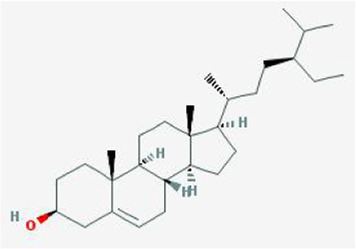	414.79	36.91	0.75
MOL000359	sitosterol	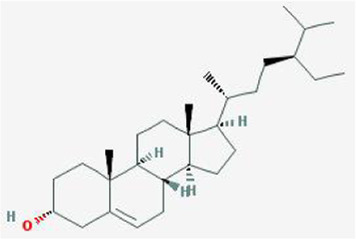	414.79	36.91	0.75
MOL004440	Peimisine	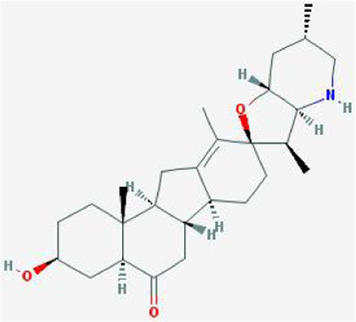	427.69	57.4	0.81
MOL009027	Cyclopamine	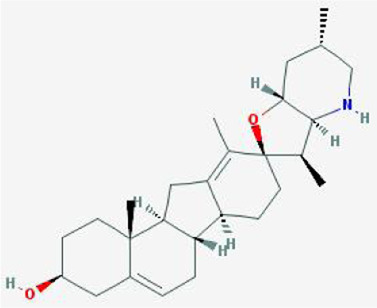	411.69	55.42	0.82
MOL009572	Chuanbeinone	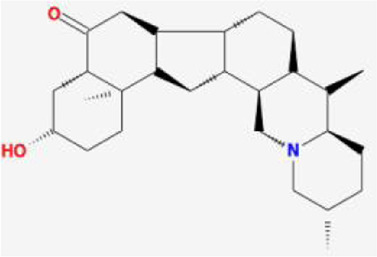	413.71	41.07	0.71
MOL009579	ent-(16S)-atisan-13,17-oxide	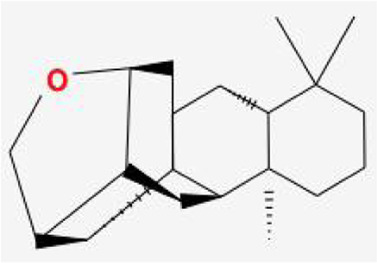	288.52	47.74	0.43
MOL009586	isoverticine	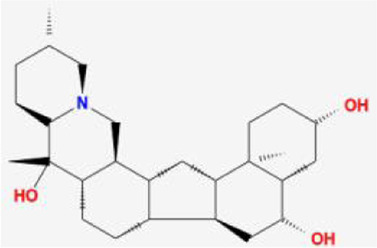	431.73	48.23	0.67
MOL009588	Korseveriline	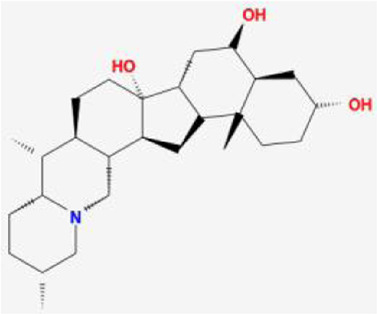	431.73	35.16	0.68
MOL009589	Korseverinine	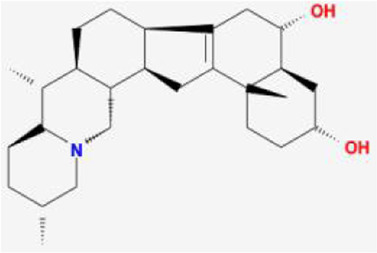	413.71	53.51	0.71
MOL009593	verticinone	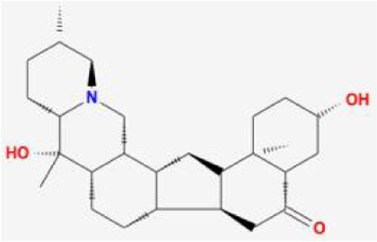	429.71	60.07	0.67
MOL009596	sinpemine A	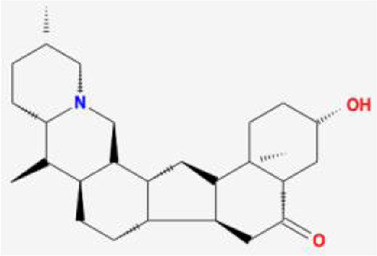	413.71	46.96	0.71
MOL009599	songbeinone	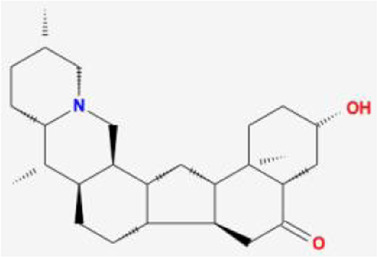	413.71	45.35	0.71

MS, Molecular structure; MW, molecular weight; OB, oral bioavailability; DL, Drug-likeness.

### 3.2 “Drug-active ingredient-core target” network construction

**FIGURE 3:**
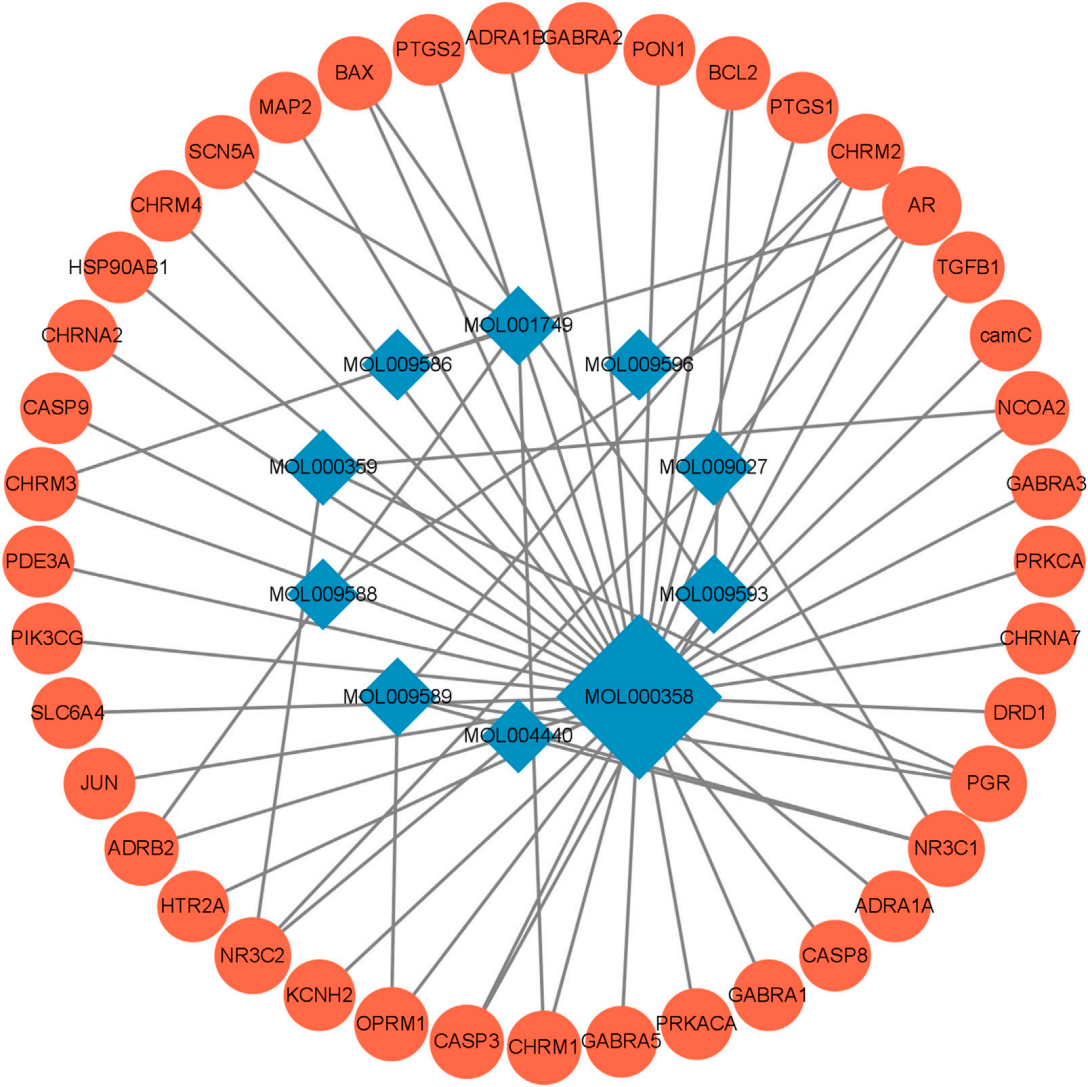
Compound-Target Network. The blue diamond represents the active compound contained in FIB. The orange circle represents the target.

The FIB active ingredients and its potential action targets were introduced into the Cytoscape 3.9.1 software to build a “drug-active ingredient-core target” network diagram, as shown in Figure 3. The blue diamond node represents the active ingredients of FIB, and the orange round node represents the potential targets of its active ingredient. The node size is related to its degree valuewhich means the greater the degree value, the larger the node. Each edge represents the interaction between the active ingredients and the action targets. The target network graph was analyzed by network topology, involved 51 nodes and 61 edges. The active components with higher degree values were bis [(2R) - 2-ethylhexyl] benzene-1,2-dicarboxylate, beta sitosterol, Korseveriline and vertiginone. Their degrees were 38, 4, 4, and 4, respectively. This network reflected the comprehensive regulation characteristics of FIB with multiple components, multiple targets, and multiple pathways, and with unique curative effect mechanisms and advantages.

### 3.3 PPI network analysis

**FIGURE 4:**
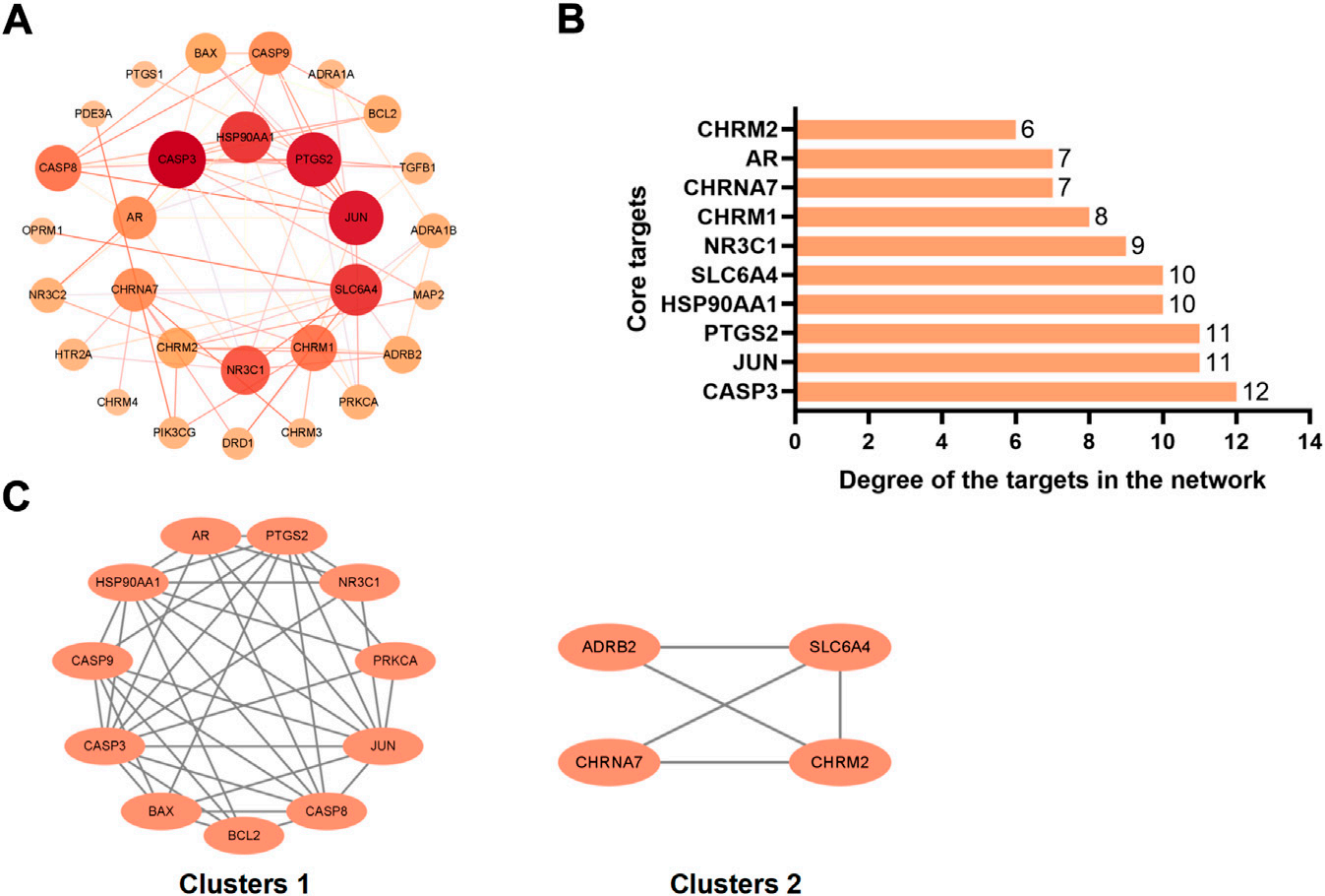
Interaction network diagram of FIB target proteins against Ischemic Strokes. PPI network of 29 duplicate targets **(A)**. Top 10 targets ranked by the degree value **(B)**. PPI network was clustered into two clusters **(C)**.

We have analyzed the protein-protein interaction of common targets of FIB and ischemic strokes through STRING database, hidden 2 connectionless nodes (PON1, SCN5A) which involved a total of 31 nodes and 78 edges. The network owned an average node degree of 5.03 and an average local clustering coefficient of 0.612. By importing the obtained data into Cytoscape 3.9.1 software, analyzing the networkto obtain the degree centrality (DC), betweenness centrality (BC) and closeness centrality (CC) of each node in the network. Selecting the nodes with DC > 5, BC > 8.893939, CC >0.500159485, and obtain 10 core targets for FIB to treat ischemic strokes (Figure 4A). CASP3, PTGS2, JUN, SLC6A4, and HSP90AA1 rank the top five in terms of degree value, indicating that these targets may be the key targets for FIB to treat ischemic strokes (Figure 4B). Cluster network analysis was performed on the PPI network using the MCODE plugin of Cytoscape (Figure 4C). The PPI network is divided into two clusters: Clusters 1 have 11 nodes and 39 edges; Clusters 2 have 4 nodes and 5 edges. Both clusters contain the above 10 core targets. Therefore, cluster network analysis confirms the screening results of hub network on core targets.

### 3.4 Go analysis

**FIGURE 5:**
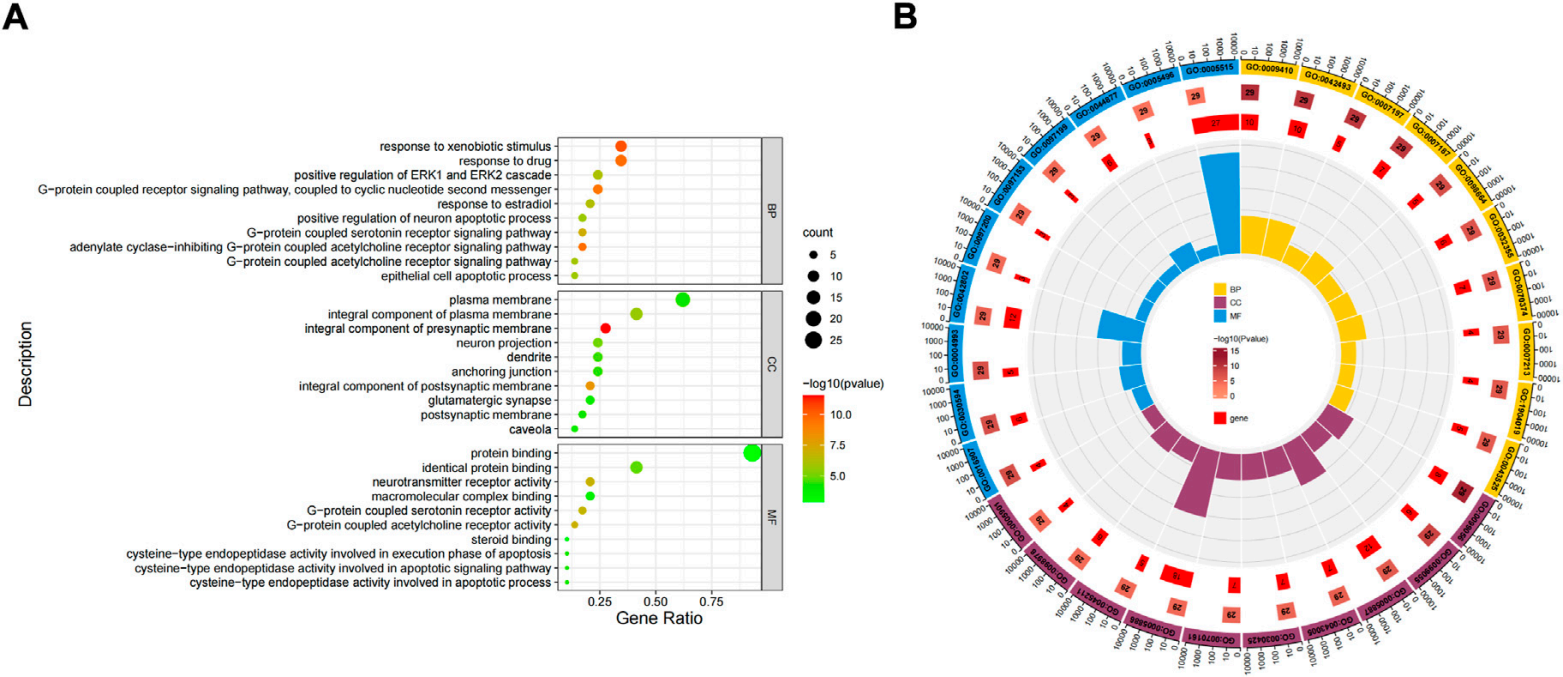
Gene Ontology (GO) function enrichment analysis of FIB in the treatment of Ischemic Strokes. Enrichment diagram of BP, CC, and MF terms **(A)**. The cluster analysis of high correlation GO terms **(B)**.

The potential role is further clarified by enrichment analysis of intersection targets. GO function enrichment analysis of 31 differential genes through DAVID database (Figures 5A,B). There were 165 related items on biological process (BP), including response to xenobiotic stimulus, response to drug, adenylate cyclase-inhibiting G-protein coupled acetylcholine receptor signaling pathway, etc. There were 37 related entries on closeness centrality, including integral component of presynaptic membrane, integral component of postsynaptic membrane, integral component of plasma membrane, etc. There were 34 related items of Molecular Function (MF), including G-protein coupled acetylcholine receptor activity, neurotransmitter receptor activity, G-protein coupled serotonin receptor activity, *etc.*

### 3.5 KEGG analysis

**FIGURE 6:**
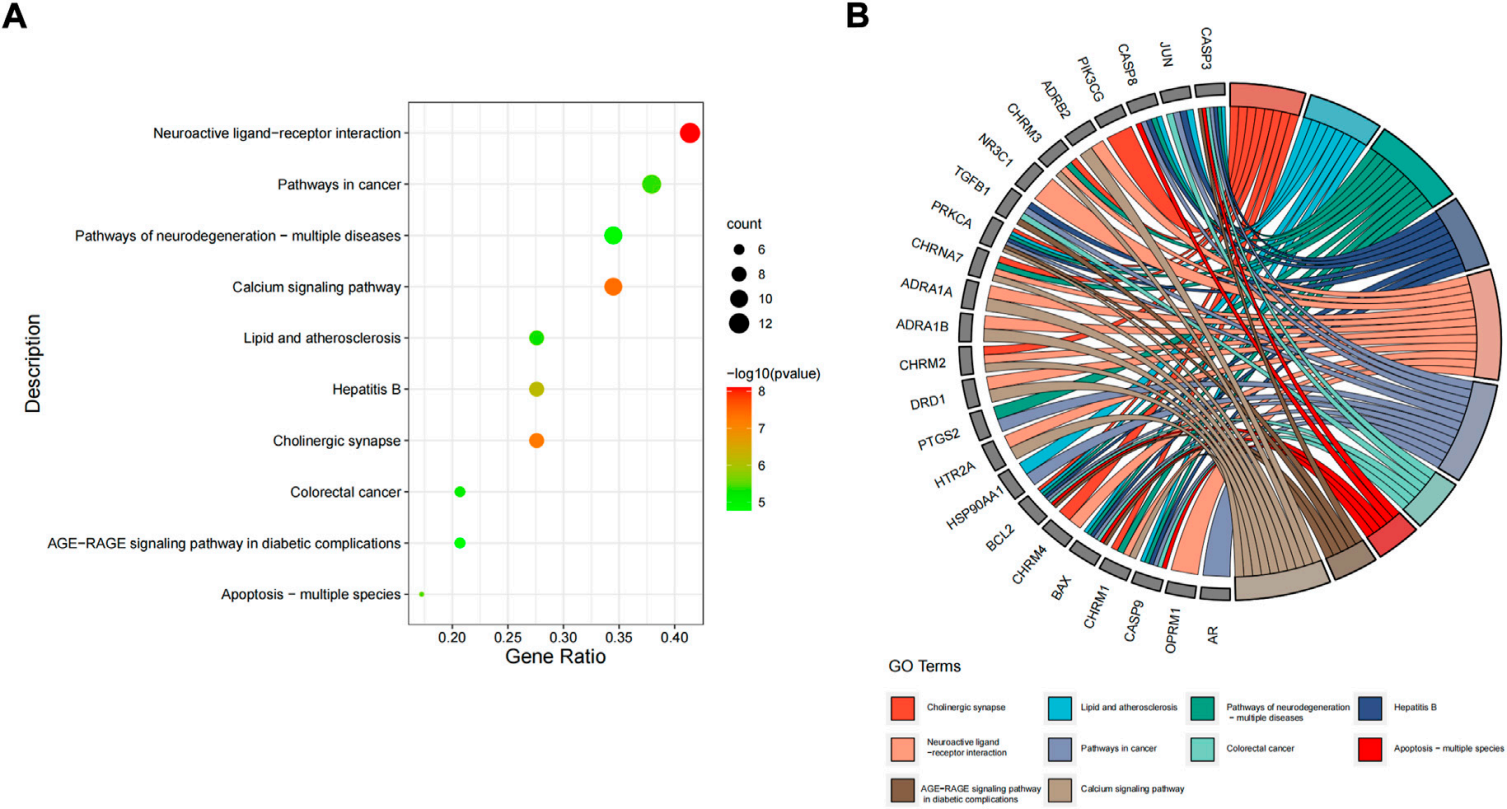
KEGG pathway enrichment analysis. The top 10 items ranked by −log10(p) value, gene count, and rich factor are shown by the bubble map **(A)**. The top 10 items ranked by −log10(p) value is shown by the Circro diagrams **(B)**.

The 31 intersecting targets of FIB and ischemic strokes were imported into the DAVID database for KEGG pathway enrichment analysis. A total of 72 pathways were acquired from the KEGG pathway enrichment analysis, which were sorted according to the -log10(p) value, and the top 10 were selected to draw a bubble map (Figure 6A) and a Circro map (Figure 6B), including Neuroactive ligand-receptor interaction, Calcium signaling pathway, Cholinergic synapse, Hepatitis B, Apoptosis—multiple species, Pathways in cancer, Lipid and atherosclerosis, Colorectal cancer, Pathways of neurodegeneration—multiple diseases, and AGE-RAGE signaling pathway in diabetic complications.

### 3.6 Network construction of “drug-active ingredient-target-disease-pathway”

**FIGURE 7:**
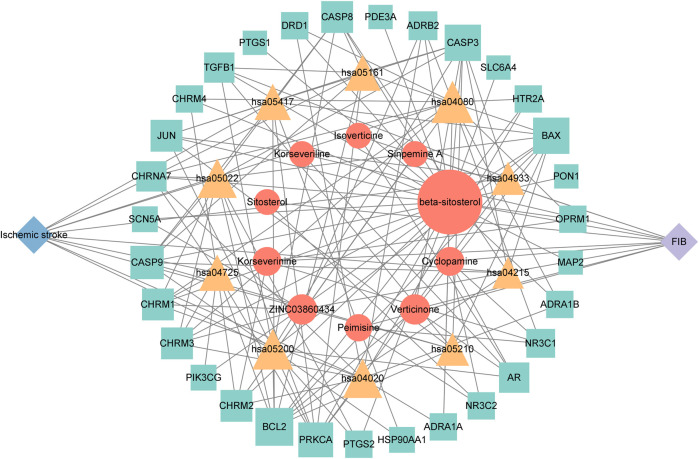
The herb-chemical composition-core target-disease-key pathway multilevel network. Pathways, compounds and targets were represented by yellow triangular-, orange circular- and green square nodes, respectively. The greater the Degree value of a node was, the larger the size were in the network.

Cytoscape 3.9.1 software was used to construct the top 10 KEGG pathways and their related target-drug-active ingredient-disease network (Figure 7). The blue diamond node is the disease name, the purple diamond node is the drug name; the orange circle node is the active ingredient of FIB, the yellow triangle node is the pathway, and the green square node is the common target of “active ingredient-disease-pathway.” The greater the degree value, the larger the node, indicating that the node is more important in the network. This network revealed the multi-component, multi-target and multi-pathway characteristics of FIB in the treatment of ischemic strokes.

### 3.7 Molecular docking

**FIGURE 8:**
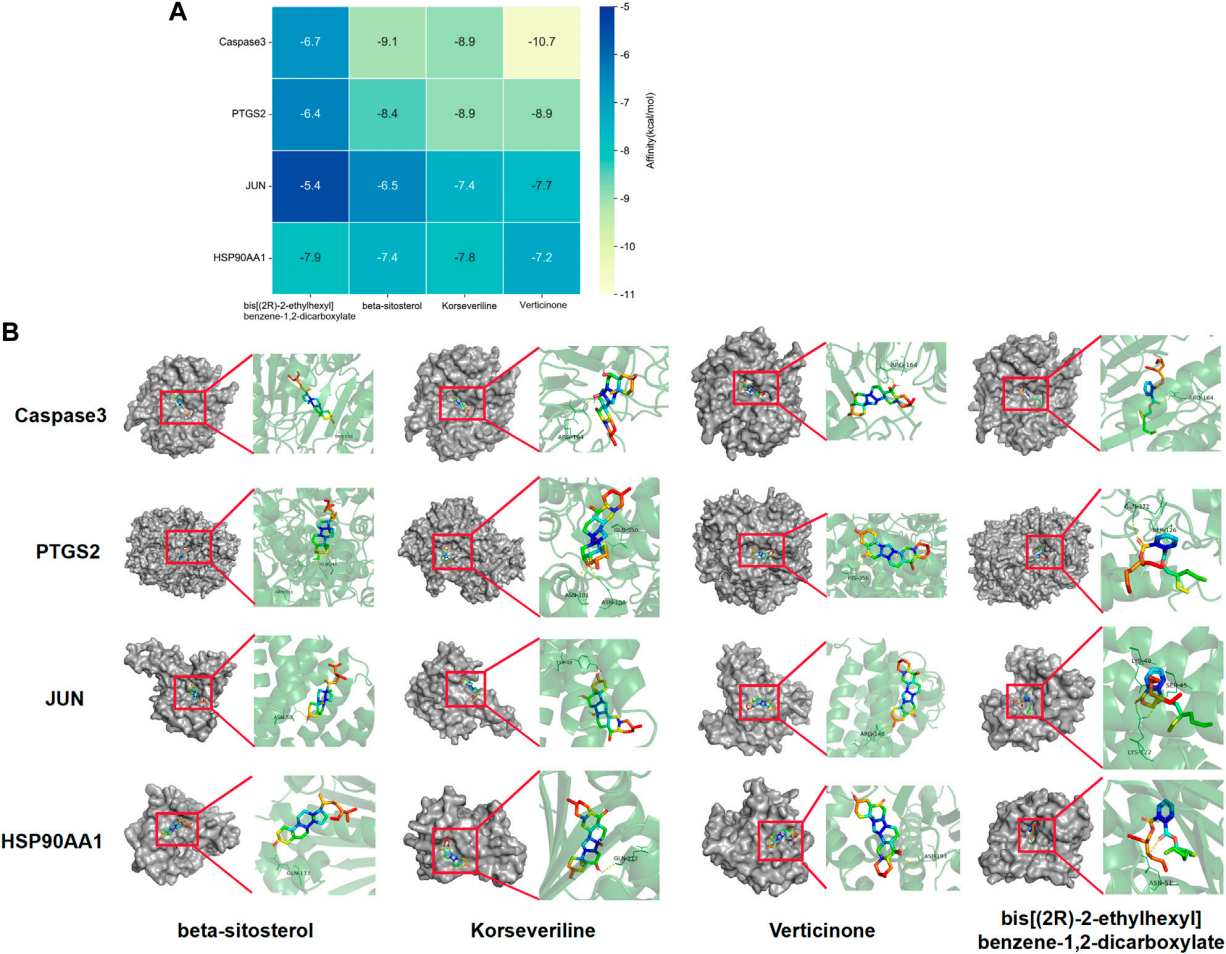
Molecular docking between core targets and active ingredients. Heat map of binding energy between four core targets and top four active ingredients by molecular docking **(A)**. Conformation examples between four core targets and top four active ingredients by molecular docking **(B)**.

AutoDock software was used to dock the screened active ingredients (bis[(2R)-2-ethylhexyl] benzene-1,2-dicarboxylate、beta-sitosterol, Korseveriline, verticinone) with the core target proteins Caspase3 (PDB: 7RNB), PTGS2 (PDB: 3NT1), JUN (PDB: 6Y3V), and HSP90AA1 (PDB: 4BQG). The lower the binding energy value is, the better the binding effect between the two. Generally, the binding energy ≤ - 5.0 kJ/mol is taken as the standard. The docking results showed that all docking energies are less than - 5 kcal · mol-1, indicating that the docking is stable (Figure 8A). The visualization of the docking results is shown in Figure 8B. There were multiple hydrogen bonds between the core target proteins and FIB (yellow dotted line indicates hydrogen bonds).

### 3.8 Evaluation of MCAO model

**FIGURE 9:**
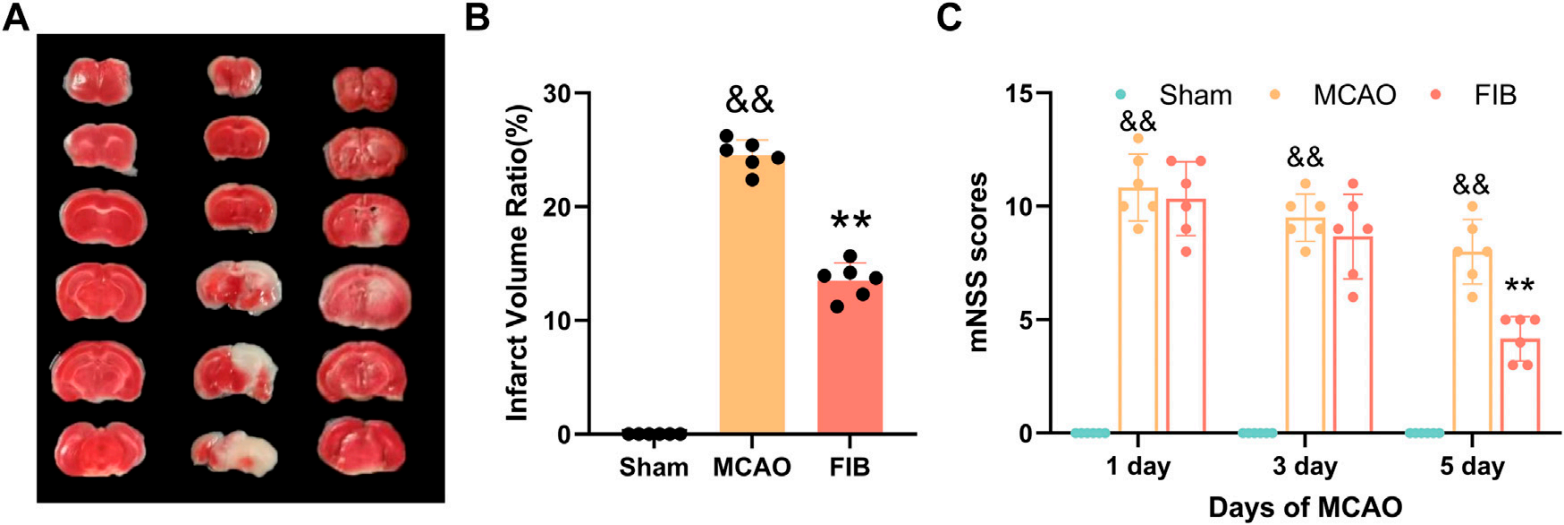
Assessment of I/R Injury Model. TCC staining of brain tissue to measure the infarct volume of brain tissue **(A,B)**. Neurological deficit score of mouse in each group after 5 days of FIB treatment **(C)**.

In order to evaluate the effect of FIB on brain nerve injury, we performed TCC staining of brain tissue to measure the infarct volume of brain tissue. No obvious lesions were found in the sham group. Compared with the Sham operation group, the infarct volume of brain tissue in the MCAO group increased significantly (*p* < 0.01). Compared with MCAO group, the infarct volume of brain tissue in FIB group was notably reduced (Figures 9A,B) (*p* < 0.01). In order to explore the effect of FIB on neural function defect of MCAO, we performed mNSS score on mice after the last administration (Figure 9C). As shown in Figure 8, the mNSS of mice in the MCAO group was remarkably higher than that in the Sham group (*p* < 0.01), but the brain mNSS of mice in the MCAO group was significantly improved after FIB treatment (*p* < 0.01).

### 3.9 Changes of core target levels in I/R injured mice treated with FIB

**FIGURE 10:**
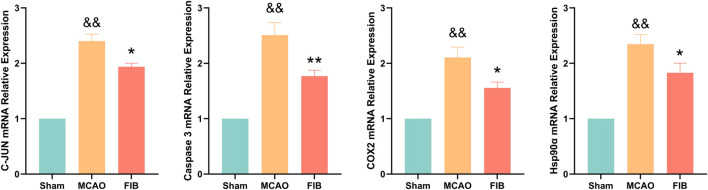
The effect of FIB on the expression levels of Caspase3, HSP90AA1, C-JUN and COX2 in MCAO mice was detected by PCR. ^&&^ *p* < 0.01, vs. the sham group. *p < 0.05, **p < 0.01, vs. the MCAO group (*n* = 3).

**FIGURE 11:**
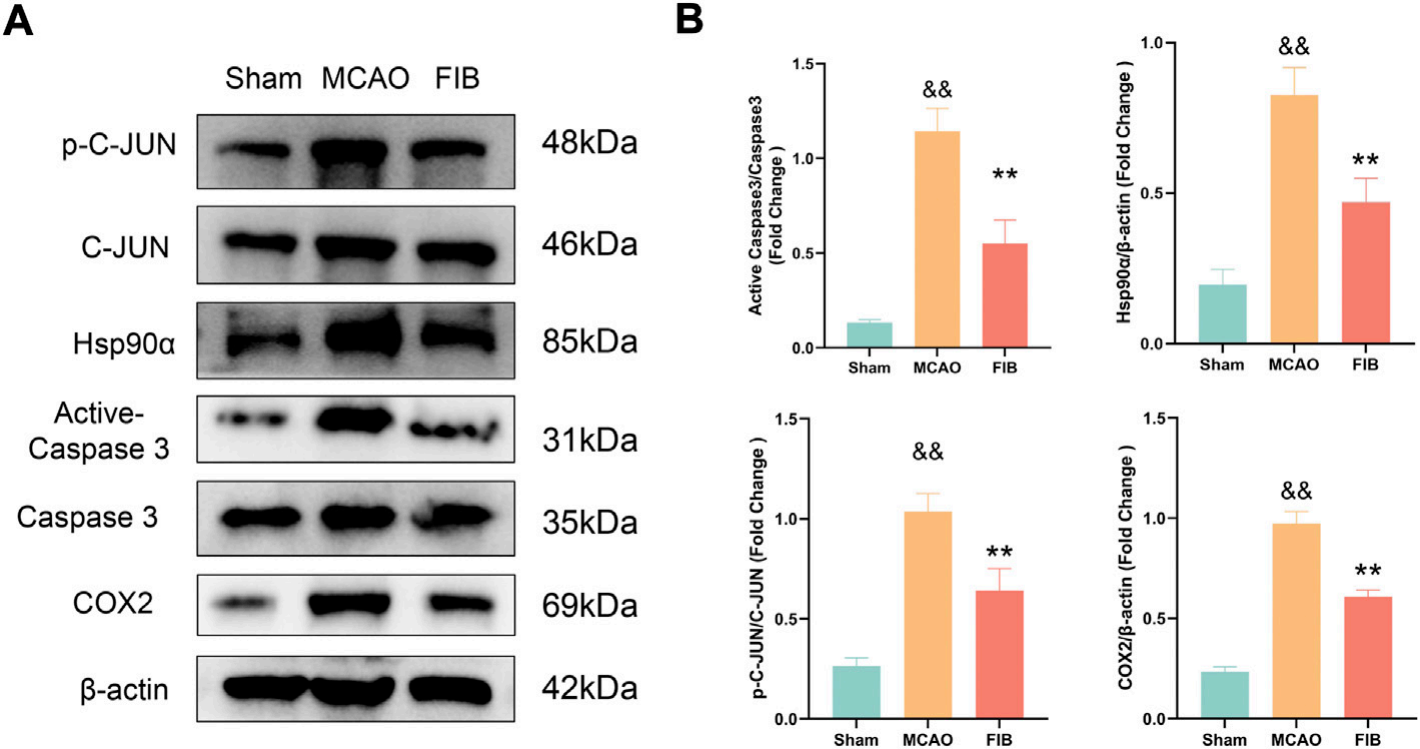
The effect of FIB on the expression levels of Caspase3, HSP90AA1, C-JUN and COX2 in MCAO mice was detected by Western Blot **(A and B)**. ^&&^ *p* < 0.01, vs. the sham group. *p < 0.05, **p < 0.01, vs. the MCAO group (*n* = 3).

We found that compared with the sham operation group, the levels of Caspase3, HSP90AA1, C-JUN, and COX2 in the model group were significantly increased by RT-qPCR (*p* < 0.01). Compared with the MCAO group, the levels of Caspase3, HSP90AA1, C-JUN and COX2 in the FIB group were significantly decreased (*p* < 0.01) (Figure 10). Then we further verified the changes of core target levels by Western Blot (Figure 11). The results presented that compared with the sham group, the levels of active-Caspase3, HSP90AA1, phosphorylated C-JUN and COX2 in MCAO group were significantly increased (*p* < 0.01). Compared with MCAO group, the levels of active-Caspase3, HSP90AA1, phosphorylated C-JUN and COX2 in the FIB group were significantly decreased (*p* < 0.01). In conclusion, FIB has a significant protective effect on ischemic stroke through the regulation of relevant core targets, which further verifies the prediction of network pharmacology.

## 4 Discussion

At present, the overall prevalence of ischemic stroke is increasing year by year, and it emerges a development trend of rejuvenation. The current basic treatment strategy for stroke is to facilitate cerebral blood perfusion and neuro-protection. Nevertheless, the limited time window and high surgical cost of thrombolytic therapy restrict its clinical application (Zhou et al., 2018). Furthermore, ischemia reperfusion therapy may lead the secondary damage to the patient’s ischemic areas and the pathogenesis of ischemic stroke is complex. Once ischemic stroke occurs, ischemia and hypoxia, inflammation, apoptosis, immune abnormalities, oxidative stress and other reactions will occur rapidly, and eventually lead to ischemic stroke and its clinical manifestations of the patients show diverse results (Kirmani et al., 2012; Chamorro et al., 2016). Therefore, treatments which targeted a single signaling pathway or target may not be effective enough. It is of great significance to study deeply the pathogenesis of ischemic stroke, explore effective treatment strategies, and develop new effective therapeutic drugs that can be applied in the clinical treatment of ischemic stroke.

Traditional Chinese medicine emphasizes individualized treatment under the guidance of a “holistic view.” Meanwhile, the stroke prevention and treatment drugs represented by traditional Chinese medicine have the advantages of “multi-component, multi-target, and multi-effect.” In addition, various components in traditional Chinese medicine can act individually or synergistically to enhance the therapeutic effect, which possess unique advantages and promising application prospects in the prevention and treatment of ischemic stroke. The application of network pharmacology to the research of traditional Chinese medicine can explore the material basis of pharmacodynamics and the mechanism of action in the treatment of diseases from the perspective of molecular network regulation, which plays an essential role in promoting and guiding the research of traditional Chinese medicine (Zhang et al., 2019). The potential mechanism of FIB in the prevention and the treatment of Ischemic Strokes were elementarily explored through large database mining, network pharmacological analysis, molecular docking technology and *in vitro* cell experiments. The effective components and action targets of FIB were screened by network pharmacological methods, and the intersection of FIB and Ischemic Strokes related targets were visually analyzed to build a drug component disease target network. Through the analysis of protein interactions in the network, the core target community was screened. The main pathways of core targets involved in the treatment of Ischemic Strokes were studied through bioconcentration analysis, and a target pathway network diagram was constructed. Finally, the four core target proteins of FIB for the treatment of Ischemic Strokes were analyzed by molecular docking. Thus, the main pathway, target protein and core components of FIB in the treatment of Ischemic Strokes can be obtained. Subsequently, an animal model of transient middle cerebral artery occlusion was established to verify the analysis of the therapeutic effect of FIB by network pharmacology.

The network pharmacology analysis found that 13 active ingredients in FIB may be the major core components in the treatment of ischemic strokes. After filtering out the common targets of FIB and Ischemic Strokes, 31 targets were screened for constructing the PPI network, and the top four core targets (CASP3, PTGS2, JUN, and HSP90AA1) were finally determined. Recent studies have shown that the activation of caspase-3 is a key link in neuronal apoptosis after the Ischemic Strokes, which will induce the formation of apoptotic bodies and the occurrence of apoptosis by cutting off contact with surrounding cells (Uzdensky, 2019). Inhibitors of caspase-3 can significantly reduce neuronal damage after experimental stroke and prolong the therapeutic window of stroke (Lee et al., 2018). Moreover, neuronal death in cerebral ischemia mainly produced excitotoxicity due to the activation of the c-Jun N-terminal kinase (JNK) pathway (Borsello et al., 2003). The results of caspase3 and c-Jun play a huge role in promoting the study of stroke pathological mechanisms and new neuroprotective strategies, indicating that FIB may be a new drugs of acting neuron protection by targeting caspase-3 and c-Jun. Most of the ischemic strokes are caused by arterial occlusion due to thromboembolism, and the common source of embolism is atherosclerosis of the great arteries. A report from the American Heart Association in 2022 pointed out that atherosclerosis is a vital risk factor for stroke (Tsao et al., 2022). Most basic researches and clinical trials have proved that selective COX-2 inhibitors can enhance the process of atherosclerosis and prevent the occurrence of ischemic strokes (Grosser et al., 2006; Yi et al., 2017). Therefore, FIB might facilitate the function of vascular wall cells and stabilize plaque to protect brain tissue by targeting COX-2. Additionally, after the occurrence of Ischemic Strokes, the damaged blood brain barrier can promote the infiltration of inflammatory cells and further activation of glial cells, and aggravate the cascade process of inflammatory reaction and ischemic brain damage (Huang et al., 2020). Studies have shown that Hsp90 inhibitors can reduce the permeability of the blood brain barrier and prevent the destruction of the endothelial barrier induced by H_2_O_2_ (Uddin et al., 2021). FIB might provide new possibilities for the treatment of diseases related to blood brain barrier dysfunction. Molecular docking was used to detect the binding affinity between typical core compounds and central targets. The results showed that bis [(2R) - 2-ethylhexyl] benzene-1,2-dicarboxylate, beta sitosterol, Korseveriline and verticine had good binding capability (affinity less than - 5 kcal/mol) to the four target proteins. In order to verify the analysis of network pharmacology, we established a tMCAO model in mice and then performed FIB treatment. The results showed that after FIB treatment, the infarct volume of brain tissue was significantly reduced, and the levels of active-Caspase3, HSP90AA1, phosphorylated C-JUN, and COX2 were enormously reduced. To sum up, FIB may protect the brain tissue after ischemia-reperfusion by alleviating the vasomotor function of cerebral vessels, improving the process of atherosclerosis, inhibiting cell necrosis and apoptosis after cerebral ischemia-reperfusion, and changing the structure of the blood brain barrier after reperfusion.

The results of GO enrichment analysis showed that the protection of FIB on ischemic strokes were realized by simultaneously activating multiple biological processes, cell components and molecular functions. The targets and the signal pathways often play a role in coordination with each other. Through the enrichment analysis of the KEGG pathway of the intersection target, 72 signal pathways were obtained, most of which exist in the nerve, inflammation, circulation and other aspects. Neuroactive ligand receptor interaction signal pathway is the collection of all receptors and ligands related to intracellular and extracellular signal pathways on the plasma membrane (Lauss et al., 2007). Ca^2+^ was not only a crucial chemical involved in neurotransmitters, but also a key chemical in the release of intercellular signals in the nervous system which was also involved in neuronal activity and the immune system (Gibson and Peterson, 1987). American researchers found that astrocytic release of extracellular mitochondrial particles was mediated by a calcium-dependent mechanism involving CD38 and cyclic ADP ribose signaling (Hayakawa et al., 2016). Alpha7 nicotinic acetylcholine receptor widely exists in central nervous system and immune system, and plays a key role in synaptic plasticity of neurons. It is not only anessencial target for improving cognitive function, but also a necessary “switch” for activating cholinergic anti-inflammatory pathway (Noviello et al., 2021). These results coincided with the enrichment analysis results of GO and KEGG. Of course, there are numerous possible targets and mechanisms of FIB, and it is uncertain whether it can have a significant effect on each node. However, we can conduct in-depth research based on the main mechanisms currently known by Ischemic Strokes, the targets and pathways contained in FIB that play an important role in the aforementioned molecular mechanisms.

## 5 Conclusion

Based on the analysis method of network pharmacology, and combined with the prediction of active ingredients and targets, GO and KEGG enrichment analysis, molecular docking, animal experiments, etc., the molecular mechanism of FIB in the treatment of ischemic strokes were predicted and verified from a systematic perspective. FIB has a favorable therapeutic effect on Ischemic Strokes by influencing multiple targets and multiple related pathways. This also provides quite a few references for our subsequent exploration of the mechanism. In the future research, we will conduct more in-depth cell and animal experiments to further study the potential mechanism of FIB in the treatment of ischemic strokes, so as to ensure the reliability of the research results. In terms of clinical research, we will design a multi-center large-sample randomized controlled study to further observe the therapeutic effect and safety of FIB on Ischemic Strokes. In conclusion, FIB is expected to become a new drug for reducing cerebral infarction area, improving neurobehavioral capability, and anti-oxidative stress after ischemic stroke.

## Data Availability

The original contributions presented in the study are included in the article/supplementary material, further inquiries can be directed to the corresponding author.
